# Metabolic shift induced by synthetic co-cultivation promotes high yield of chain elongated acids from syngas

**DOI:** 10.1038/s41598-019-54445-y

**Published:** 2019-12-02

**Authors:** Martijn Diender, Ivette Parera Olm, Marten Gelderloos, Jasper J. Koehorst, Peter J. Schaap, Alfons J. M. Stams, Diana Z. Sousa

**Affiliations:** 1 Laboratory of Microbiology, Wageningen University & Research, Stippeneng 4, 6708 WE Wageningen, the Netherlands; 2 Laboratory of Systems and Synthetic Biology, Wageningen University & Research, Stippeneng 4, 6708 WE Wageningen, the Netherlands; 3 Centre of Biological Engineering, University of Minho, Campus de Gualtar, 4710-057 Braga, Portugal

**Keywords:** Environmental biotechnology, Applied microbiology, Microbial communities

## Abstract

Bio-catalytic processes for sustainable production of chemicals and fuels receive increased attention within the concept of circular economy. Strategies to improve these production processes include genetic engineering of bio-catalysts or process technological optimization. Alternatively, synthetic microbial co-cultures can be used to enhance production of chemicals of interest. It remains often unclear however how microbe to microbe interactions affect the overall production process and how this can be further exploited for application. In the present study we explored the microbial interaction in a synthetic co-culture of *Clostridium autoethanogenum* and *Clostridium kluyveri*, producing chain elongated products from carbon monoxide. Monocultures of *C. autoethanogenum* converted CO to acetate and traces of ethanol, while during co-cultivation with *C. kluyveri*, it shifted its metabolism significantly towards solventogenesis. In *C. autoethanogenum*, expression of the genes involved in the central carbon- and energy-metabolism remained unchanged during co-cultivation compared to monoculture condition. Therefore the shift in the metabolic flux of *C. autoethanogenum* appears to be regulated by thermodynamics, and results from the continuous removal of ethanol by *C. kluyveri*. This trait could be further exploited, driving the metabolism of *C. autoethanogenum* to solely ethanol formation during co-cultivation, resulting in a high yield of chain elongated products from CO-derived electrons. This research highlights the important role of thermodynamic interactions in (synthetic) mixed microbial communities and shows that this can be exploited to promote desired conversions.

## Introduction

The rapid increase in world population and consequent pressure on the environment, together with the accelerated depletion of fossil reserves, urges measures for the implementation of waste-to-cradle technologies. Thermal gasification followed by the conversion of the generated syngas to fuels and chemicals is one of the routes that can play role in future circular economy. The advantage of this route is that a broad spectrum of recalcitrant and low biodegradable carbon waste streams (e.g. lignin, municipal waste) can be ultimately converted into fuels and chemicals^1–3^. One of the main components of syngas, carbon monoxide (CO), is also present in off-gases produced in heavy industry (e.g. steel mill industry), and syngas fermentation technologies can be easily extrapolated for the depuration and valorization of these polluted streams.

In the last years efforts have been made to improve syngas fermentation and expand its scope of products. This includes development of genetic tools for acetogenic clostridia^4–6^, bioreactor development^7^ and the study of the physiology of the syngas biocatalysts^8–10^. Autotrophic Clostridia (e.g. *Clostridium autoethanogenum, Clostridium ljungdahlii*) are potential biocatalysts for syngas conversion, employing acetogenic/solventogenic metabolism, generating acetate and ethanol as main products. Because of the overall limited amount of naturally produced compounds by these autotrophs, efforts have been made to expand the scope of products from syngas fermentation. Next to genetic engineering approaches^11^, there is increasing attention for alternative cultivation strategies, involving application of defined and undefined mixed cultures^12–15^. The defined co-cultivation approaches have previously been shown to be efficient in expanding the scope of products from gas fermentation towards chain elongated acids and longer alcohols^12,13^. In the constructed food webs, a carboxydotrophic microorganism (i.e. able to use CO) generates acetate and ethanol as an intermediate product allowing a second organism performing reverse β-oxidation to utilize these products and form chain-elongated acids. Additionally, chain elongated alcohols were found, resulting from the further reduction of the respective acids by the carboxydotroph. Co-cultures were very stable during subsequent transfers^13^ and chemostat runs^12^, indicating both strains endured naturally in the process, and seemed to benefit from each other during growth. Understanding the type of interactions that take place in these defined co-cultures is essential for engineering and improving these defined co-cultures for better performance. So far it remained unclear how the strains influence each other during growth and how product formation can be optimized. In order to uncover the interaction between the strains, this study focused on uncovering the response of *C. autoethanogenum* to environmental changes induced by *C. kluyveri* via chemostat cultivation and transcriptomics analysis. The results indicate that the main interaction is driven by thermodynamics rather than genetic regulation, showing that thermodynamics plays an essential role in product formation in (synthetic) mixed microbial communities.

## Results

Experiments were performed in continuous stirred-tank reactors (CSTR) to accurately control environmental conditions (pH, temperature, stable medium composition) and microbial growth rates. Operating conditions of CO/syngas-fed bioreactors are summarized in Table 1. Gas consumption efficiency varied between 60 and 95%, depending on the conditions, and differences are possibly explained by slight changes in reactor setup or variations in total gas feeding rate.

**Table 1:** Operating conditions of CO/syngas-fed bioreactors and identification of experiments performed of each run. Reactors were operated at pH 6.2 and were gas transfer limited.

Run nr.	CO inflow (mmol/l/d)	H_2_ inflow (mmol/l/d)	Additional medium components	Strain	HRT (d)	Volume liquid (l)	CO consumption efficiency (%)	Experiment
1	155	0		CA	1.5	0.75	90	Transcriptome
	155	0		CA + CK	1.5	0.75	90	Transcriptome
2	155	77		CA	1.5	0.75	84	Transcriptome
	155	77	0–8 mM butyrate	CA	1.5	0.75	84	Transcriptome Butyrate addition
3	116	0–93		CA	2	1	80–95	H_2_ addition
	116	0–128		CA + CK	2	1	80–95	H_2_ addition
4	155	0	0–90 mM acetic acid	CA	1.5	0.75	60–70	Acetate addition
	155	0	0–90 mM acetic acid	CA + CK	1.5	0.75	60–70	Acetate addition

HRT = hydraulic retention time.

CA = *C. autoethanogenum*, CK = *C. kluyveri*.

### *C. autoethanogenum* alters product spectrum during co-cultivation without altering transcription of genes involved in central carbon and energy metabolism

**Figure 1:**
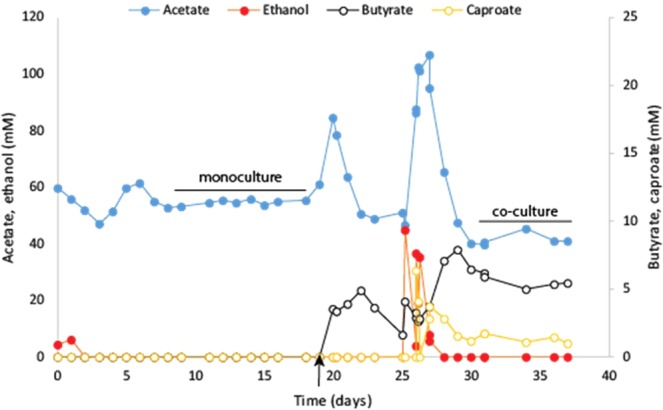
Monoculture vs. co-culture growth in chemostat (reactor run 1). Monoculture of *C. autoethanogenum* was grown from day 0–19, and co-cultivation initiated at day 19 (black arrow) and operated till day 37. Left and right axis are indicated using closed or open symbols respectively. The culture was spiked with ethanol at day 25 to temporarily boost *C. kluyveri* growth. Lines labelled ‘monoculture’ or ‘co-culture’ indicate the time frame where transcriptomics samples were taken (at least 3 samples in each condition).

A monoculture of *C. autoethanogenum* was inoculated in the CSTR and fed with solely CO, at constant pH of 6.2 and 37 °C, for 20 days. After this period, the reactor was inoculated (5% v/v) with a culture of *C. kluyveri* for the establishment of the synthetic co-culture (under similar environmental conditions) (Table 1, run 1). In monoculture, *C. autoethanogenum* produced 54.7 ± 0.9 mM acetate and low amounts of ethanol (~0.2 mM) in steady state (Fig. 1, days 9–19). After inoculation of *C. kluyveri* and establishment of the co-culture, butyrate and caproate were formed with an average product concentration of 5.5 ± 0.7 mM and 1.3 ± 0.3 mM, respectively (Fig. 1, days 30–37). In the co-culture, acetate levels stabilized at 41.7 ± 2 mM and ethanol was detected in trace amounts (<0.1 mM). Carbon monoxide consumption rates in both mono- and co-culture conditions were similar: 147 ± 0.3 mmol l^−1^ d^−1^ vs. 151 ± 1.2 mmol l^−1^ d^−1^, respectively. Biomass levels during the mono- and co-culture steady state conditions were around 0.60 and 0.42 g/l respectively. In monoculture, electrons were mainly directed to acetogenesis as almost all electrons in the end-product were situated in acetate. In the co-culture 21% of the electrons end up in butyrate and 8% in caproate. Based on chain elongation reaction stoichiometry, 1.2% of the total electrons found in end-products in the co-culture were transferred to protons by *C. kluyveri*, resulting in hydrogen formation. Assuming the chain elongation stoichiometry shown in Eqs. 1 and 2, it is possible to estimate the production of acetate (45 mM) and ethanol (7 mM) by *C. autoethanogenum* in co-culture. Compared to monoculture conditions, a clear displacement of electrons towards the production of ethanol is observed in co-culture. This metabolic shift between the mono- and co-culture condition suggests that *C. kluyveri* drives the metabolism of *C. autoethanogenum* towards solventogenesis. Microorganisms were not observed to aggregate or form macro-structures during co-cultivation, as confirmed by visual and microscopic observation.

1 $$6\,{{\rm{C}}}_{2}{{\rm{H}}}_{5}{\rm{OH}}+4\,{{\rm{CH}}}_{3}{{\rm{COO}}}^{-}\to 5\,{{\rm{C}}}_{3}{{\rm{H}}}_{7}{{\rm{COO}}}^{-}+{{\rm{H}}}^{+}+3\,{{\rm{H}}}_{2}{\rm{O}}+2\,{{\rm{H}}}_{2}$$

2 $$6\,{{\rm{C}}}_{2}{{\rm{H}}}_{5}{\rm{OH}}+5\,{{\rm{C}}}_{3}{{\rm{H}}}_{7}{{\rm{COO}}}^{-}\to 5\,{{\rm{C}}}_{5}{{\rm{H}}}_{11}{{\rm{COO}}}^{-}+{{\rm{CH}}}_{3}{{\rm{COO}}}^{-}+{{\rm{H}}}^{+}+3\,{{\rm{H}}}_{2}{\rm{O}}+2\,{{\rm{H}}}_{2}$$

Comparative transcriptome analysis of the mono- and co-culture in steady state shows no major up- or down-regulation of genes involved in central carbon and energy metabolism of *C. autoethanogenum*. Genes of the Wood-Ljungdahl pathway in *C. autoethanogenum* are highly expressed in both the mono- and co-culture. The expression of Wood-Ljungdahl pathway genes does not show an uniform response to co-cultivation, suggesting no genetic regulatory response is taking place (Table 2, Supplementary Data I). Additionally, the significant changes observed are minimal and do not exceed a 2–3 fold change in most cases. Genes related to alcohol metabolism in *C. autoethanogenum*, such as alcohol dehydrogenase (adhE, CAETHG 3747–3748) and acetaldehyde oxidoreductase (aor, CAETHG 0092, 0102), are not differentially expressed in mono- and co-culture. During co-cultivation a cassette of genes, CAETHG 2642–3064, is upregulated 5–10 fold, coding for genes related to amino acid synthesis, pili/flagella formation/assembly and antibiotics production. Furthermore, the most striking changes in the *C. autoethanogenum* transcriptome were observed in the metal import related genes for iron and molybdenum. A relatively strong downregulation was observed for iron-transport related genes: feoA/B and several iron-chelate importers (Table 2). Additionally, genes identified as molybdenum transporters were downregulated between 10 and 80-fold. Also increase in genes identified as ferric iron regulator (fur) transcription factors, reported to act as an iron import repressor^16^, are increasingly expressed in co-culture matching with the downregulation in expression for iron importers. Determining iron concentrations levels in the medium gave a concentration of 1.13 ± 0.24 µM in the monoculture compared to 1.76 ± 0.30 µM in the co-culture.

**Table 2:** Highlights of significant changes (p < 0.01) in the comparative transcriptome of *C. autoethanogenum* in monoculture vs. *C. autoethanogenum* in co-culture with *C. kluyveri*.

	Locus tag	Gene	Fold change (up/down)
Wood-Ljungdahl pathway	CAETHG 2789–2799	Formate dehydrogenase (fdhA/hytA-E)	1–6 (↑)
	CAETHG 1618	Formate-THF ligase	1.4 (↓)
	CAETHG 1614–1615	methylene-THF reductase	1.9 (↓)
	CAETHG 1617	Methenyl-THF cyclohydrolase	1.4 (↓)
	CAETHG 1610–1611	CODH/ACS	1.5–2 (↓)
Alcohol metabolism	CAETHG 1813, 1841	Alcohol dehydrogenase	2–3 (↓)
	CAETHG 1819, 1830	Acetaldehyde dehydrogenase	3–4 (↓)
Redox metabolism	CAETHG 1580	Nfn-complex	2 (↓)
	CAETHG 3003–3005	CODH	1.5–2.5 (↑)
	CAETHG 3840–3841	Hydrogenase	8–11 (↓)
Other	CAETHG 2642–3064	Host defence and assimilatory metabolism	5–10 (↑)
	CAETHG 0252–0254, 3479–3481	Iron transporters (feoA/B)	50–100 (↓)
	CAETHG 3827–3830, 0088–0092, 2677–2679	Iron transporters (iron-chelate)	5–10 (↓)
	CAETHG 0313–0315, 0671–0672, 3822–3825	molybdenum transporters	10–80 (↓)
	CAETHG 0018, 1463, 2706	Ferric uptake regulator (fur)	2–7 (↑)

### Additional hydrogen feeding triggers solventogenesis in *C. autoethanogenum*, but does not influence its transcriptome

In order to assess the effect of main products of *C. kluyveri* (hydrogen and butyrate) on *C. autoethanogenum*, a monoculture of *C. autoethanogenum* was grown on 155 mmol l^−1^ d^−1^ CO and 77 mmol l^−1^ d^−1^ H_2_ in presence or absence of 8 mM butyrate (Table 1, run 2). Addition of hydrogen resulted in production of acetate (58 ± 0.8 mM) and ethanol (6.5 ± 0.5 mM) (Fig. S1). Compared to growth on only CO, where all the electrons were used for acetogenesis (Fig. 1, day 9–19), feeding both CO and H_2_ resulted in 85% of the electrons in acetate and 15% into ethanol. Experiments using a feed of solely CO at a rate similar to the combined H_2_/CO feed rate (~232 mmol l^−1^ d^−1^) resulted in solely acetate formation in steady state. When 8 mM butyrate was fed in addition to H_2_ no additional changes were observed and the physiological profile did not significantly change compared to when no butyrate was added (Fig. S1).

Growth on CO:H_2_ compared to growth on solely CO, did not result in large differences in expression of genes from the Wood-Ljungdahl pathway (CAETHG 1608–1621) (Supplementary Data II). Most of the genes were ~2 fold higher expressed in the condition with CO/H_2_, except for the bifurcating formate dehydrogenase, not being differently expressed. Interestingly, the CODH not related to ACS (CAETHG 3003–3005), was upregulated when H_2_ was present (~10 fold). The alcohol metabolism does not show a uniform response as there was no clear up- or downregulation trend in acetaldehyde dehydrogenases, acetaldehyde oxidoreductases and alcohol dehydrogenases when exposed to CO/H_2_. Expression of hydrogenases appears not to change by presence of hydrogen. A block of genes (CAETHG 1705 – CAETHG 1912) related to assimilatory metabolism was slightly downregulated by ~3–6 fold. Overall this suggests a minor response to hydrogen addition on the overall transcriptome, with no major changes of genes involved in the energy or central carbon metabolism. In the condition with butyrate, no further changes were observed in the transcriptome (Supplementary Data III). Thus, presence of hydrogen or butyrate appear not to trigger any large changes in transcriptome, not explaining the metabolic shift towards ethanol. Transcriptomic changes that are observed with hydrogen addition to monocultures of *C. autoethanogenum* are also not similar to the transcription response of *C. autoethanogenum* in the co-culture condition.

### Hydrogen addition promotes chain elongation in co-culture via stimulation of *C. autoethanogenum* metabolism

**Figure 2:**
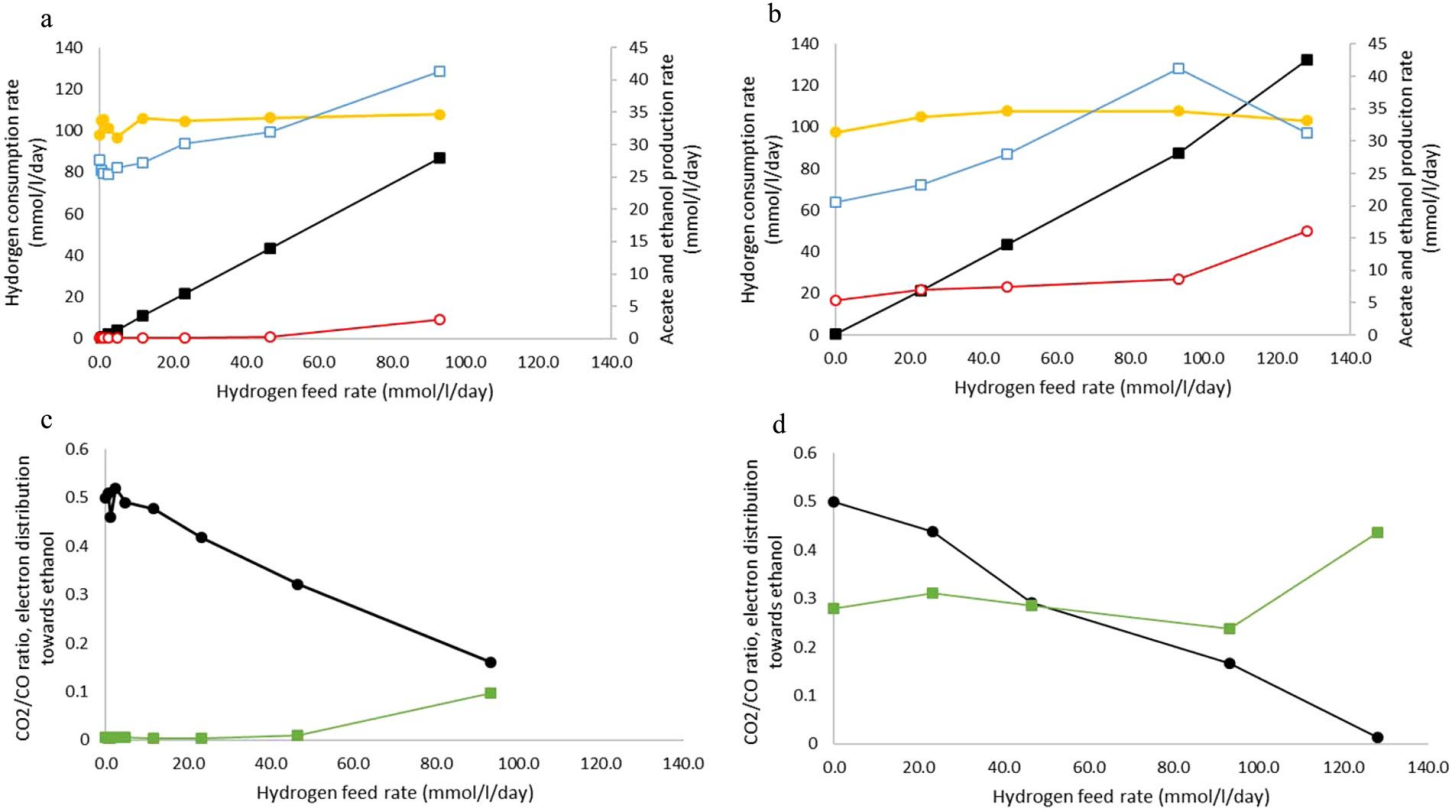
Physiological profile of *C. autoethanogenum* when grown on 116 mmol/l/day CO with increasing hydrogen feed in monoculture (**a/c**) or co-culture (**b/d**). In case of co-culture, ethanol and acetate production, and the derived electron distribution are reverse calculated from observed chain elongation activity (Table 3). Left and right axis are indicated using closed or open symbols respectively. (**a/b**) Hydrogen consumption rate (black squares), CO consumption rate (yellow circles), acetate production (blue open squares) and ethanol production rate (red open circles). (**c/d**) CO_2_/CO ratio (black circles), electrons directed towards ethanol by *C. autoethanogenum* (green squares).

The observation that addition of hydrogen during carboxydotrophic growth causes a shift towards solventogenesis in *C. autoethanogenum*, suggests that hydrogen exchange between *C. kluyveri* and *C. autoethanogenum* could be a possible trigger for the observed metabolic shift during co-cultivation. In the co-culture grown on CO only, hydrogen was produced at a rate of 0.2 mmol l^−1^ d^−1^, while predicted hydrogen production rates derived from chain elongation activity are in the range of 2 mmol l^−1^ d^−1^_._ This suggests that hydrogen produced by *C. kluyveri* was indeed consumed by *C. autoethanogenum*. In order to test at which H_2_ feed rates solventogenesis is triggered in *C. autoethanogenum*, a gradient of H_2_ inflow with a steady CO background was created (Table 1: run 3). Hydrogen inflow rates from 0 to 93 mmol l^−1^ d^−1^ were tested in addition to 116 mmol l^−1^ d^−1^ CO. Hydrogen consumption was observed for all tested inflow concentrations and was linearly correlated with the influx (Fig. 2a). The CO_2_/CO ratio decreased linearly with increasing hydrogen inflow (Fig. 2c). Acetate formation increased gradually with increasing hydrogen influx and stimulation of solventogenesis was not observed at the tested influx rates below 46.6 mmol l^−1^ d^−1^ (Table 3, Fig. 2a). At higher hydrogen inflow rates, a stable ethanol production rate was observed in steady state conditions up to 3 mmol l^−1^ d^−1^ when fed with 93 mmol l^−1^ d^−1^ of hydrogen. In this case electrons in ethanol account for ~9% of the electrons in total found in products (Fig. 2c), not meeting the 20–30% observed in the co-culture solely fed with CO (Fig. 2d).

**Table 3:** Steady state concentrations of fermentation products and cellular biomass in the mono- and co-culture during the experiments with supplementation of hydrogen and acetate supply.

	Hydrogen feed rate (mmol/l/day)	Acetate (mM)	Ethanol (mM)	Butyrate (mM)	Caproate (mM)	Biomass (g_dry weight_/L)
monoculture	0	56.0	0.2			0.28
	23	61.0	0.2			0.22
	47	65.0	0.4			0.24
	93	83.0	6.0			0.3
co-culture	0	35.5	<0.1	5.5	1.7	0.22
	23	39.5	<0.1	6.9	2.3	0.22
	47	48.5	<0.1	7.6	2.4	0.23
	93	73.2	<0.1	10.0	2.2	0.23
	128	48.0	5.5	12.6	7.1	0.23
	**Acetate feed rate** **(mmol/l/day)**	**Acetate** **(mM)**	**Ethanol** **(mM)**	**Butyrate** **(mM)**	**Caproate** **(mM)**	**Biomass** **(g**_**dry weight**_**/L)**
monoculture	0	40.7	0.20			0.15
	16	63.2	0.26			0.14
	33	81.1	0.55			0.09
	60	119.7	1.12			0.08
co-culture	0	23.4	<0.1	5.3	1.8	0.13
	16	33.5	<0.1	7.7	4.0	0.15
	33	40.0	<0.1	10.1	5.0	0.14
	60	68.0	<0.1	14.0	5.5	0.14

In order to test if hydrogen would enhance productivity and efficiency of the co-culture, a range of H_2_ from 0 to 128 mmol l^−1^ d^−1^ was fed to the co-culture. With increasing hydrogen inflow, chain elongation activity was stimulated compared to conditions where no hydrogen was added (Table 3). When reverse calculating acetate and ethanol production rates, an increase in both acetogenic and solventogenic rate by *C. autoethanogenum* was observed (Fig. 2b). The electron distribution towards ethanol remained between 26 and 30% (Fig. 2d), being similar to the co-culture growing solely on CO. This pattern was not observed when supplying excess of hydrogen (128 mmol l^−1^ d^−1^), causing depletion of CO_2_. This resulted in a rapid increase in solventogenic activity shuttling 45% of the electrons towards ethanol (Fig. 2d). This resulted in a background of 5.5 mM net ethanol formation, showing ethanol formation by *C. autoethanogenum* was no longer the limiting factor in the co-culture (Table 3).

### External acetate addition promotes ethanol as electron sink and enhances chain elongation in co-culture

**Figure 3:**
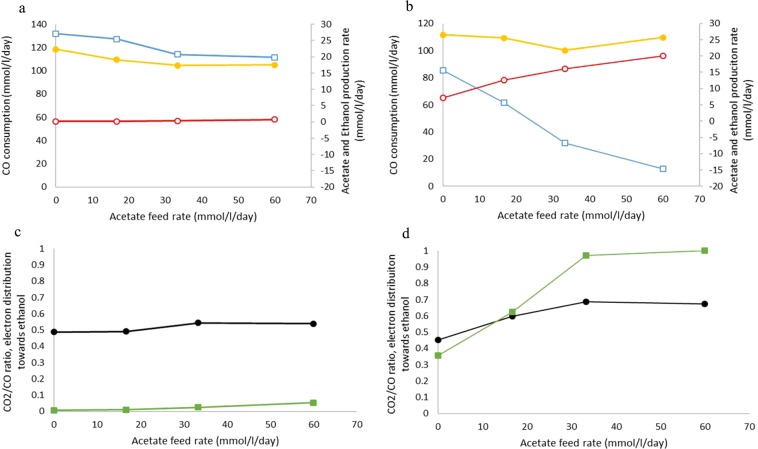
Physiological profile of *C. autoethanogenum*when grown on 155 mmol/l/day CO with increasing acetate feed in monoculture (**a/c**) or co-culture (**b/d**). In case of co-culture, ethanol and acetate production, and the derived electron distribution are reverse calculated from observed chain elongation activity (Table 3). Left and right axis are indicated using closed or open symbols respectively. (**a/b**) CO consumption rate (yellow circles), acetate production (blue open squares) and ethanol production rate (red open circles). (**c/d**) CO_2_/CO ratio (black circles), electrons directed towards ethanol by *C. autoethanogenum*(green squares).

The effect of acetate in steering the metabolism of *C. autoethanogenum* towards solventogenesis was tested; increasing acetate concentrations in a range of 0 to 60 mmol l^−1^ d^−1^ (Table 1: run 4). Ethanol production rates went up from 0 to 0.75 mmol l^−1^ d^−1^ (Fig. 3). Despite that the activity of acetogenesis decreased from 27 ± 0.7 mmol l^−1^ d^−1^ to 20 ± 3 mmol l^−1^ d^−1^ at an acetate inflow of 60 mmol l^−1^ d^−1^, the majority of electrons (~95%) were still moved towards acetogenesis (Fig. 3c). Biomass levels decreased when more acetate was provided, illustrating the negative effect of increased acetic acid concentrations on *C. autoethanogenum* (Table 3), confirming results from earlier studies^10,17^.

Supplying similar amounts of acetate to the co-culture resulted in an increase of chain elongated products up to 14 mM butyrate and 5.5 mM caproate, in steady-state (Table 3). Reverse calculating the production rate of ethanol and acetate in these conditions, showed that the acetate production decreases and ethanol production increases at increasing acetate feed rate (Fig. 3b). At 60 mmol l^−1^ d^−1^ acetate inflow, *C. autoethanogenum* was fully solventogenic, causing all CO derived electrons to be used for ethanol production (Fig. 3d). This contrasts with the 5% electrons in ethanol obtained in monoculture at the same acetate feed rate (Fig. 3c). All acetate produced by *C. autoethanogenum* was further converted to ethanol, causing net conversion of externally supplied acetate to ethanol. In contrast to the monoculture, we observed no decrease in biomass when external acetate was added to the co-culture, remaining stable around 0.2 g/l in all tested conditions (Table 3).

## Discussion

*C. autoethanogenum* and *C. kluyveri* can grow in co-culture and produce chain-elongated products from CO or syngas^13^. In the chemostat experiments described here, total biomass during co-cultivation of *C. autoethanogenum* and *C. kluyveri* was lower (or similar) to biomass of *C. autoethanogenum* in monoculture, indicating that *C. autoethanogenum* has a higher biomass yield when not in co-culture. This can partly be explained by the observed metabolic shift of *C. autoethanogenum* from acetogenic (monoculture) to more solventogenic (co-cultivation with *C. kluyveri*). The energy yield per CO consumed of the production of acetate is overall higher compared to the production of ethanol^9^, explaining less biomass formation during co-cultivation. Additionally, based on the transcriptomes of mono- and co-cultures, there is an apparent stress response of *C. autoethanogenum* to *C. kluyveri*. Some anabolic pathways (e.g. amino acid synthesis) and host defense systems (e.g. sporulation, flagella/pili) increase expression in *C. autoethanogenum* upon co-cultivation, likely contributing to higher maintenance costs. Additionally, chain-elongated acids produced by *C. kluyveri* could exert some toxicity towards *C. autoethanogenum* due to their hydrophobic properties and likely result in increased maintenance costs^18^. The lower biomass concentration of *C. autoethanogenum* in the co-culture subsequently explains the downregulation in expression of iron and molybdenum metal importers observed (10–100 fold). Less trace elements are required for biomass build-up, increasing their concentration in the medium. This is supported by the higher concentrations of iron found in the co-culture medium compared to the monoculture. A potential role for these metal transporters in the metabolic shift to ethanol production is unlikely. If metal transporters were to play a role in ethanol formation a decrease in their expression would also be expected in the H_2_/CO fed condition (where ethanol formation is also increased), however this is not the case.

Chain-elongation activity observed in mixed communities fed with H_2_/CO_2_ or in bio electrodes are assumed to be the result of cross feeding of intermediate products (e.g. ethanol /lactate)^19^. Similarly, growth of *C. kluyveri* in the co-culture described here is expected to be dependent on the acetate and ethanol provided by *C. autoethanogenum*. While these metabolites are expected to be the main intermediates in the co-culture, use of other intermediate electron shuttles, such as metals, pili or proteins, cannot be ruled out. However, *C. kluyveri* was shown not to directly interact with electrodes in electrochemical cells^20^, and only slight interaction of *C. kluyveri* with electron shuttles (e.g. 2-hydroxy−1,4-naphthoquinone, ferrocyanide, methyl-viologen) was noted. This was however found not to be related to reverse β-oxidation, and growth of *C. kluyveri* on these mediators without addition of ethanol and acetate was not possible^20^. We assume that ethanol is the most likely electron donor for reverse β-oxidation in this co-culture of *C. autoethanogenum* and *C. kluyveri* for two reasons: (i) ethanol concentrations during co-cultivation are lower (<0.1 mM) than in monocultures (~0.2 mM) (Table 3); and, (ii) any other form of electron transfer (e.g. pili/electron transfer protein) would require the formation of butyryl-CoA from acetate by *C. kluyveri* to act as donor for the chain elongating process, implying a large ATP investment due to required reversal of acetate kinase.

In co-culture experiments, *C. kluyveri* keeps ethanol concentrations low, resulting in a more suitable condition for ethanol production by *C. autoethanogenum*. Several studies suggest that acetogenic/solventogenic metabolism of gas fermenting acetogens might be controlled by thermodynamics rather than by gene expression^10,17^. In accordance with this, we did not observe large changes on the expression of genes involved in energy and central carbon metabolism of *C. autoethanogenum* in mono-/co-culture. Addition of butyrate (main product of *C. kluyveri*) to cultures of *C. autoethanogenum* did not stimulate solventogenesis, ruling out a potential stimulatory effects of butyrate on ethanol production. In addition to butyrate, small amounts of H_2_ are formed by *C. kluyveri* in the chain elongation process; in the co-culture experiments performed here a production of 2 to 7 mmol l^−1^ d^−1^ H_2_ is estimated (~4% of the electrons passing through the *C. kluyveri* metabolism). Net hydrogen production rates in the co-cultures were in the range of 0.2 mmol l^−1^ d^−1^, indicating that H_2_ formed by *C. kluyveri* is consumed by *C. autoethanogenum*. Utilization of hydrogen by *C. kluyveri* was assumed not to take place, due to the described inhibitory effect of H_2_ on *C. kluyveri* chain elongation metabolism^21^, and the observed efficient hydrogen removal by *C. autoethanogenum* (Fig. 2). In line with what is reported by others^17^, addition of H_2_ to *C. autoethanogenum* monocultures did steer its metabolism towards solventogenesis, but only when supplied in sufficient amounts (>46 mmol l^−1^ d^−1^) (Fig. 2). Still, ethanol production in H_2_-supplemented monocultures was still significantly lower than in co-cultures (Fig. 2). In view of this, while the H_2_ produced by *C. kluyveri* might contribute to the metabolic shift towards ethanol, it is unlikely to be the sole explanation for it.

During acetate addition experiments, it is observed that ethanol formation is promoted while acetogenesis is inhibited (Fig. 3). By equating the formulas for Gibbs free energy of formation for acetic acid and ethanol formation, the concentration of ethanol can be calculated where its production is still thermodynamically feasible (Eqs. 3, 4). When grown solely on CO (Eq. 3) the ethanol concentration at which ethanol production is still feasible depends linearly on acetic acid concentration and quadratically on the CO concentration in the liquid. An inverse quadratic relation with ethanol formation for CO_2_ is found, indicating CO_2_ depletion should be beneficial for solventogenic activity. Feeding with a mixture of CO and H_2_, assuming utilization ratio according to feed composition, a mix between CO and hydrogen metabolism will occur. Ethanol concentrations can be estimated for CO:H_2_ ratios between 1:0 and 1:1 using Eq. 4. As long as ethanol concentrations remain below the thermodynamic threshold concentration, it remains favorable to be produced.

3 $$[Ethanol]=\frac{{[CO]}^{2}\cdot [Acetic\,acid]}{{[C{O}_{2}]}^{2}}\cdot {e}^{\frac{\Delta {{G}^{0}}_{acetogenesis}-\Delta {{G}^{0}}_{solventogenesis}}{RT}}$$

4 $$[Ethanol]=\frac{[Acetic\,acid]\cdot {[CO]}^{2-2x}\cdot {[{H}_{2}]}^{2x}}{{[C{O}_{2}]}^{2-2x}}\cdot {e}^{\frac{\Delta {{G}^{0}}_{acetogenesis}-\Delta {{G}^{0}}_{solventogenesis}}{RT}}{\rm{where}}\,x=\frac{[CO]}{[CO]+[{H}_{2}]}$$

**Figure 4:**
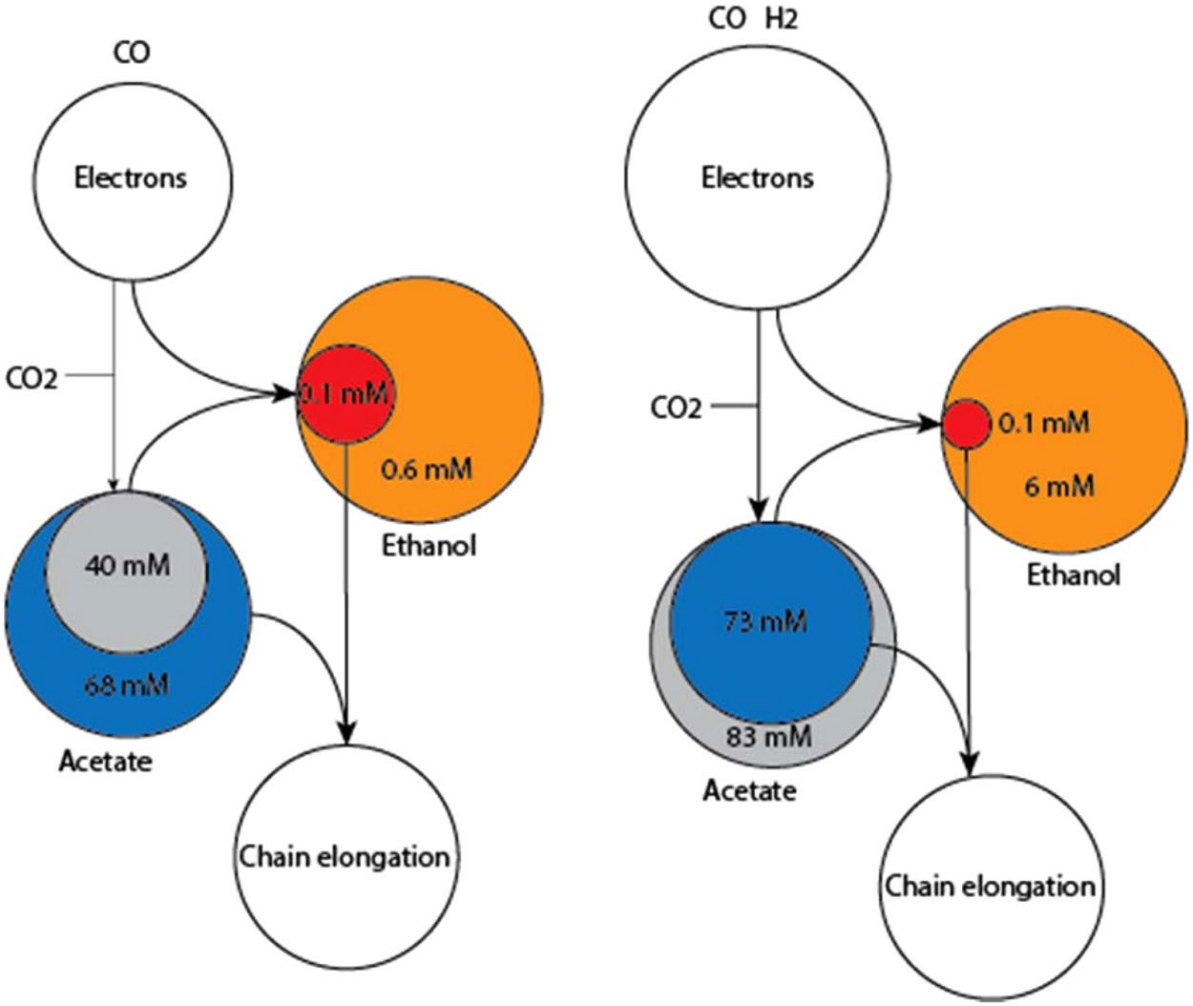
Quantitative model for the preferred pathways to deposit electrons during acetate feeding at 60 mmol l^−1^ day^−1^ (left) and hydrogen feeding at 93 mmol l^−1^ day^−1^ (right). Blue and red spheres indicate the measured concentration of the compound in the co-culture (*C. autoethanogenum*+ *C. kluyveri*) whereas grey and orange spheres indicate the corresponding monoculture (*C. autoethanogenum*) steady state concentrations. A smaller pool in the co-culture suggests pathways towards this pool are more feasible while a larger pool in co-culture suggests pathways to be less feasible compared to monoculture conditions. Flux through the pathways is indicated via arrows.

Steady state concentrations of acetate and ethanol in the monoculture of *C. autoethanogenum* were 38 and ~0.2 mM, respectively (Table 3). Under similar conditions, the co-culture generates acetate and ethanol concentrations of 23 and <0.1 mM, respectively (Table 3). This lower ethanol concentration, caused by continuous ethanol removal by *C. kluyveri*, potentially keeps reduction of acetate to ethanol favorable as electron sink for *C. autoethanogenum* (resulting in a higher ethanol balance, Fig. 4). When providing external acetate, the thermodynamic ethanol threshold concentration is elevated (Eq. 3/4) as is observed from increasing ethanol concentrations in the monoculture, while simultaneously making acetogenesis increasingly unfavorable (Fig. 3a).

In contrast to the acetate feeding experiment, hydrogen addition boosted overall chain-elongation activity in the co-culture, but did not cause *C. autoethanogenum* to shift towards solventogenesis on top of the initial shift, keeping the fraction of electrons ending-up in ethanol between 26 and 30% (Fig. 2d). Hydrogen addition stimulates both acetogenesis and solventogenesis (Fig. 2a), and is likely the cause for the absence of a further relative shift towards ethanol production. Only during excessive hydrogen feeding, resulting in CO_2_ limitation, a further shift towards solventogenesis is observed (Fig. 2d). CO_2_ limitation likely lowers the feasibility of acetogenesis as electron sink, making solventogenesis more favorable (Eq. 3/4). CO_2_ limitation might also be the reason why hydrogen feeding only has an effect on solventogenesis in monocultures of *C. autoethanogenum* at higher feeding rates, as more CO_2_ is captured from the environment (Fig. 2c).

The observations made in this study, suggest the metabolic shift observed in *C. autoethanogenum* is regulated by thermodynamics. However, a metabolic shift based on change in protein levels or post-translational modifications cannot be ruled out. But, based on previous indications that the acetogenic syngas metabolism is steered by thermodynamics rather than genetic regulation^10,17^, the absence of major and clear unidirectional changes in transcriptome of the central carbon and energy metabolism (Table 2), and the observations that the metabolism of *C. autoethanogenum* can be strongly steered by changing the concentration of its end products (Figs. 2, 3), are strong indications that the metabolic shift observed in this co-culture is the result of thermodynamics.

The observation that the metabolism of *C. autoethanogenum* can be strongly steered by changing the environmental concentrations of its end products poses an interesting concept for production potential of *C. autoethanogenum* and similar strains. Continuous ethanol removal from the fermentation broth by subsequent reactions or extraction can pull the metabolism of *C. autoethanogenum* towards solventogenesis, and could improve the yield of the process significantly. The observation that *C. autoethanogenum* produced solely ethanol in co-culture during the acetate stimulation experiment (Fig. 3d) is interesting as in monocultures of *C. autoethanogenum* the ratio of ethanol/acetate is in most studies reported to be <1. Several cases report ethanol/acetate ratios >1^17,22–24^, however these are usually observed in the pH range 4–5^23^ and under relatively high gas feed rates^24^. This is in contrast to the relatively ‘high’ 6.2 pH, low gas feed system presented here where eventually ~100% solventogenic conditions for *C. autoethanogenum* are achieved. From an application perspective these results are interesting as in the co-culture conditions relatively more electrons are utilized for ethanol production, overall increasing the yield of chain elongated products per gaseous substrate consumed. This is further promoted in the case of external acetate addition to co-cultures fed with CO, resulting in acetate reduction to ethanol and in a high yield of chain elongated acids. The thermodynamic interactions in this co-culture are likely difficult to mimic in monoculture and therefore synthetic co-cultures might have potential to achieve higher product/substrate efficiencies compared to monocultures. In addition, this study shows that thermodynamic interactions play an important role in the formation of the product spectrum of individual microbial components in a community. This indicates that product spectra reported for microbial monocultures might not represent the ‘natural’ production spectra of the studied strains.

## Conclusion

Co-cultivation of *C. autoethanogenum* and *C. kluyveri* results in a significant shift from acetogenesis to solventogenesis in *C. autoethanogenum*. Ethanol consumption by *C. kluyveri* is suggested to be the main driver for this, promoting the flux of electrons from CO to ethanol in *C. autoethanogenum*. In the co-culture, solventogenic activity of *C. autoethanogenum* could be further stimulated by addition of hydrogen or acetate, resulting in a stronger effect compared to the monoculture conditions. Production of solely ethanol from CO derived electrons could be obtained by *C. autoethanogenum*, but only in presence of *C. kluyveri*. Using this concept when constructing synthetic communities might be a valuable way to increase the efficiency of biobased production processes.

## Material and Methods

### Strains and cultivation

*Clostridium autoethanogenum* (DSM 10061) and *Clostridium kluyveri* (DSM 555) were obtained from the DSMZ strain collection (Deutsche Sammlung von Mikroorganismen und Zellkulturen, Braunschweig, Germany). Both strains were initially cultivated according to supplier instructions. Experiments were conducted in medium containing (per liter of medium): 0.9 g NaCl, 0.9 g NH_4_CL, 0.2 g MgSO_4_ * 7H_2_O, 0.75 g KH_2_PO_4_, 1.94 g K_2_HPO_4_ * 3H_2_O, 0.02 g CaCl_2_ and 0.5 mg resazurin. The medium was supplemented with the following trace-elements (per liter of medium): 1.5 mg FeCl_2_ * 4 H_2_O, 0.070 mg ZnCl_2,_ 0.025 mg FeCl_3_ * 6 H_2_O, 0.1 mg MnCl * 4 H_2_O, 0.006 mg H_3_BO_3_, 0.190 mg CoCl_2_ * 6H_2_O, 0.002 mg CuCl_2_ * 2 H_2_O, 0.024 mg NiCl_2_ * 6 H_2_O, 0.2 mg Na_2_WO_4_, 0.056 mg Na_2_MoO_4_ * 2 H_2_O, and 0.0035 mg Na_2_SeO_3_. The medium was boiled and cooled under N_2_ flow on ice, after which 0.75 g L-cysteine was added per liter of medium as reducing agent. The pH was set to 6 using 1 M NaOH and 1 M HCl. Medium was dispensed under N_2_ flow, into glass serum bottles that were immediately capped with rubber stoppers and aluminum caps. The headspace was filled with the desired gas, to a final pressure ranging from of 150 kPa. Bottles were autoclaved immediately after preparation. Before inoculation, the medium was further supplemented with a vitamin solution in a 1:50 dilution, containing per liter: 1 mg biotin, 10 mg nicotinamid, 5 mg p-aminobenzoic acid, 5 mg panthothenic acid, 10 mg thiamin, 25 mg pyridoxamine, 5 mg cyanocobalamine and 5 mg riboflavin. Other additives, such as: yeast extract (0.5 g/l), ethanol and acetate were added from sterile stock solutions. Unless stated otherwise, batch bottle cultivation was done at 37 °C while shaking at 150 rpm.

### Bioreactor operation

1.5 liter (total volume) bioreactors (Applikon, Delft, the Netherlands) were operated in continuous mode. Gases CO, H_2_ or N_2_ were supplied using mass flow controllers (Brooks Instruments BV, Ede, the Netherlands). The liquid volume in the reactors was set to 750 or 1000 ml. Stirring was performed by two Rushton stirrers on a single shaft, with blades placed at 33% and 66% of the liquid height. Unless specified otherwise, the pH was maintained at a value of 6.2 using 3 M KOH. Gas outflow rates were determined using a bubble counter. All mentions of gas-volumes in supply or production rates throughout the text are considered to be at 1 atm pressure and 298 K.

After sterilization, reactors were connected to the control tower, initiating temperature (37 °C) and pH control. Prior to inoculation, reactors were flushed for 3 hours with N_2_ at a rate of 20 ml/min, to create anaerobic conditions. Vitamins, yeast extract, and L-Cysteine were introduced in the reactor in the same concentration as described for bottle cultivation. Before inoculation the gas flow was switched to the desired gas feed. After reduction of the medium below −300 mV the reactor was inoculated with the culture in a 1:20 ratio. A peristaltic pump (Masterflex, Gelsenkirchen, Germany) was used to supply liquid medium to the reactors; hydraulic retention time (HRT) was set to 1.5 or 2 days (depending on the experiment). The medium tank contained complete medium and was acidified using 30 ml 37% HCl per 10 L medium to ensure sterile conditions of the inflowing medium. In experiments where acetate was provided in the inflow, 25, 50 or 90 mM acetic acid was added to the medium. The medium vessel was continuously sparged with nitrogen (5 L/h) to ensure anaerobic conditions of the inflow medium.

Reactors were inoculated with 1:20 ratio of exponentially growing *C. autoethanogenum*. In case of co-cultivation, an exponentially growing *C. kluyveri* culture was inoculated on top of *C. autoethanogenum* in a 1:20 total volume ratio.

### Analytical techniques

Liquid phase composition was determined via high pressure liquid chromatography (HPLC) installed with a MetaCarb 67 H column (Agilent Technologies, Santa Clara, CA). Operation temperature was 45 °C with a flow rate of 0.9 ml/min. Both RI and UV detector were used for detection of the compounds. Eluent was composed of 0.005 M H_2_SO_4_. Liquid samples of 0.5 ml were taken and centrifuged at 13000 *g*. Subsequently, 0.4 ml supernatant was taken and added to 0.6 ml 10 mM DMSO in 0.05 M H_2_SO_4_ solution. Concentrations below 0.1 mM could not be accurately be quantified and are further referred to as trace amounts.

In order to more accurately determine lower amounts of alcohols (<1 mM) samples were analysed on GC-2010 (Shimazu, Tokyo, Japan) equipped with an flame ionization detector. The column (DB wax UI of 30 m, 0.53 µM diameter) was operated at a temperature gradient of 40 °C for 5 minutes, subsequently ramping to 200 °C over 5 minutes and remaining at the higher temperature for 5 minutes.

For gas analysis, 0.2 ml gas samples were taken and analyzed in a Compact GC 4.0 (Global Analyser Solutions, The Netherlands). In order to measure CO and H_2_ a Molsieve 5 A column operated at 100 °C coupled to a Carboxen 1010 pre-column was used. CO_2_ was measured using a Rt-Q-BOND column operated at 80 °C. A thermal conductivity detector was used for detection in all cases.

Dry weight was determined by centrifuging a predetermined volume of culture broth (of at least 10 ml) and washing the pellet in ultrapure water two times. Cells were then dried at 120 °C in pre-weighed aluminum baskets, before re-weighing.

Iron levels were determined using a colorimetric method. Samples were centrifuged for 2 min 10000 g to pellet the cells and supernatant used for subsequent analysis. Reagents used to determine iron concentration were: A: 10 mM ferrozine in 0.1 M ammonium acetate, B: 1.4 M hydroxylamine-HCl in 2 M HCl, C: 10 M ammonium acetate (pH 9.5). A 100 µl sample (or diluted sample) was mixed with 100 µl reagent A and 800 µl MilliQ water. Sample was mixed and measured at 562 nm to determine Fe(II) content. Fe(III) content was subsequently determined by adding 187.5 µl reagent B, mixing and leaving it for 20 minutes before addition of 62.5 µl of reagent C and measuring absorbance at 562 nm.

### Transcriptomics

For transcriptomic analysis three, 25 ml samples were drawn from the chemostat operating in steady state. Samples were taken for 3 subsequent turnovers (under steady-state). Samples were collected in an anaerobic vials sealed with rubber stopper, containing 10 ml RNA later (ThermoFisher, Massachusetts, USA). After sampling, vials were instantly submerged in a slurry of dry-ice and ethanol causing instant freezing of the broth. Vials were stored at −80 °C till extraction.

After defrosting the samples on ice, RNA-extraction was performed. Cells were centrifuged at 4 °C in 50 ml Greigner tubes at 6000 g for 15 minutes. Pellets were washed with 500 µl, 20 mM TE-buffer (pH 7.2), centrifuged at 10000 g (4 °C) and re-dissolved in 150 µl TE-buffer. Cell pre-treatment was done via Lysozyme incubation for 10 min at 20 °C. Cell lysis and RNase inactivation was done by addition of a mix containing 3 μl β-mercaptoethanol, 1 µl proteinase-K and 150 µl of Gram positive lysis solution (Gram positive DNA extraction kit, Masterpure). Lysis was initiated by 10 min incubation at 60 °C, while vortexing every 5 minutes. After incubation, the mix was quickly cooled on ice and proteins precipitated using the protein precipitation mix (Gram positive DNA extraction kit, Masterpure). Debris was removed via centrifugation at 4 °C 10000 g. The sample was further cleaned and purified via the automated Maxwell LEV simply RNA extraction kit (Promega, Madison, USA), according to manufacturer’s instructions. Quality of the RNA extracts was checked using Bio-Analyser (Agilent, Santa Clara, USA), according to manufacturer’s instructions. RNA was collected in RNase free water and stored at −80 °C till further analysis. Depletion of rRNA and sequencing was performed by Novogene (Hong Kong, China) using HiSeq, paired-end reads.

### Transcriptome data analysis

Genomes of *Clostridium autoethanogenum* DSM 10061 (GCA_000484505.1) and *Clostridium kluyveri* DSM 555 (GCA_000016505.1) were retrieved from the European Nucleotide Archive. Each genome was converted to Resource Description Framework (RDF) using the SAPP conversion module (v.0.1.274)^25^. Gene expression levels were obtained via the Transcriptomics module using bowtie2 (v.2.1.0). The reads were mapped to each genome independently and to the co-culture for the identification of cross-mapping and to obtain expression levels of the experimental configurations. Read counts were retrieved using SPARQL from the genome repository and converted into an expression matrix. Differential expression analysis was performed using DeSeq. 2. The monoculture transcriptome of *C. autoethanogenum* on CO was compared to the co-culture, monoculture on CO/H_2_. Furthermore the transcriptome of *C. autoethanogenum* on CO/H_2_ was compared to the CO/H_2_ + butyrate condition.

## Supplementary information

Supplementary Figure S1

Data File S1

Data file S2

Data file S3
