# Supplementary material for: Metabolic shift induced by synthetic co-cultivation promotes high yield of chain elongated acids from syngas
Source: Sci Rep. 2019 Dec 2;9:18081. doi: 10.1038/s41598-019-54445-y (PMC6889307; doi:10.1038/s41598-019-54445-y)
Supplement: Supplementary file 1 — Supplementary Figure S1 [file 41598_2019_54445_MOESM1_ESM.pdf]

Metabolic shift induced by synthetic co-cultivation promotes high yield of chain elongated acids from syngas

Martijn Diender[1], Ivette Parera Olm[1], Marten Gelderloos[1], Jasper J. Koehorst [2], Peter J. Schaap[2], Alfons J.M. Stams[1,3] & Diana Z. Sousa[1*]

[*]Corresponding author: Diana.Sousa@wur.nl

[1] Laboratory of Microbiology, Wageningen University, Stippeneng 4, 6708 WE, Wageningen, the Netherlands.
[2] Laboratory of Systems and Synthetic Biology, Wageningen University, Stippeneng 4, 6708 WE, Wageningen, the Netherlands.
[3] Centre of Biological Engineering, University of Minho, Campus de Gualtar, 4710-057, Braga, Portugal.

Supplementary Material

Supplementary Figure S1

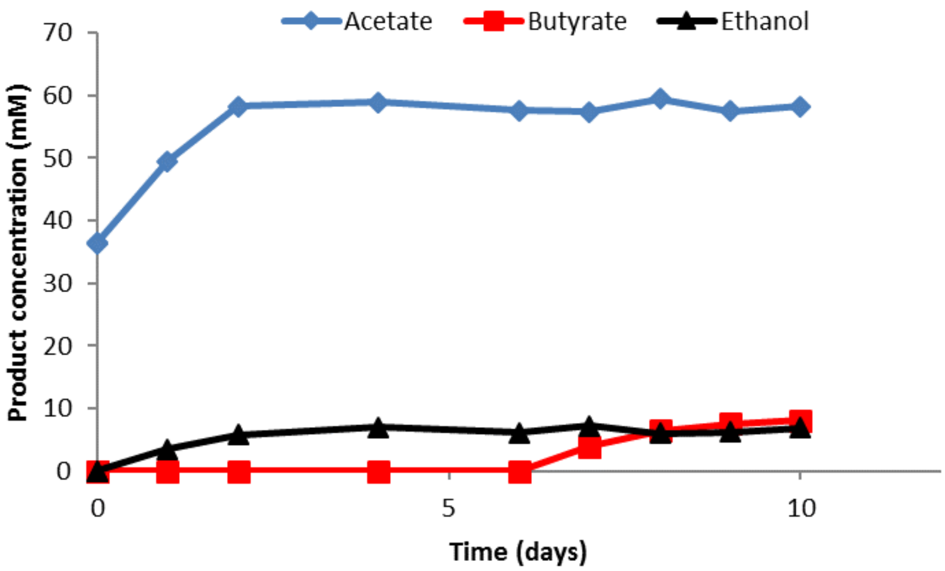

Figure S1. Production spectrum of C. autoethanogenum exposed to hydrogen or hydrogen and butyrate*. Lines labelled 'Hydrogen' or 'Hydrogen + butyrate' indicate the time frame where transcriptomics samples were taken (at least 3 samples). Butyrate was introduced after day 6.*
